# Adherence to CPAP therapy for sleep apnea in patients aged over 70 years old

**DOI:** 10.1007/s11325-021-02398-w

**Published:** 2021-06-06

**Authors:** Heidi Avellan-Hietanen, Tiina Aalto, Paula Maasilta, Oili Ask, Adel Bachour

**Affiliations:** 1 Sleep Unit, Heart and Lung Centre, Helsinki University Hospital, University of Helsinki, P. O. BOX 160, 00029 HUS Helsinki, Finland; 2 Internal Medicine and Rehabilitation, Helsinki University Hospital, University of Helsinki, P. O. BOX 340, 00029 HUS Helsinki, Finland

**Keywords:** Adherence, Daily use, Elderly, Nine-hole test, Pinch-test

## Abstract

**Purpose:**

Adherence to continuous positive airway pressure (CPAP) for obstructive sleep apnea (OSA) syndrome has not been established in patients over 70 years of age, whereas several studies have reported adherence below that age. This trial was designed to address this evidence gap.

**Methods:**

Consecutive senior (> 70 years) patients with OSA, mean respiratory event index (REI) 34/h, body mass index (BMI) 31 kg/m^2^, and junior (< 50 years) patients (REI 37/h, BMI 31 kg/m^2^) were included.

**Results:**

At year follow-up among 72 senior patients (35 women) and 71 junior patients (17 women), there was no difference in the percentage of patients abandoning CPAP (senior 47% vs. junior 43%) or in CPAP daily use (4:53 ± 2:44 hh:min vs. 4:23 ± 3:00 hh:min).

**Conclusions:**

CPAP adherence in senior patients with OSA was not poorer than that of a younger group of OSA patients. Advanced age should not be an obstacle to CPAP initiation.

## Introduction

Obstructive sleep apnea (OSA) is highly prevalent after the age of 65 years [1]. Sleep apnea leads to sleep disruption, resulting in excessive daytime sleepiness. In this manuscript, we use the term senior patient for those > 70 years old and junior patient for those < 50 years old. In senior patients, sleep apnea symptoms may be conflated with the functional impairments of aging [2]. Continuous positive airway pressure (CPAP) is an effective treatment for sleep apnea in all patients [3]. McMillan et al. also recommended CPAP therapy in senior patients suffering from OSA. They reported that CPAP reduces sleepiness and is marginally more cost-effective over 12 months than best supportive care alone.

With aging, memory may become impaired and physical dexterity decreases [4]. Memory loss may lead to reduced therapy adherence if not compensated with external aid [5]. CPAP therapy requires sufficient upper extremity mobilization and strength. We therefore believe that CPAP therapy adherence may be poorer in seniors compared to junior patients due to memory loss, weakness in upper extremities, and reduced dexterity.

The proportion of senior individuals among the general population is increasing in developed countries [https://www.statista.com/statistics/521152/population-of-finland-by-age/]. CPAP adherence in the community is generally poor, with rates ranging from 65 to 88% [6–8]. CPAP daily use in senior patients was reported by McMillan [2] to be very low at 1 year, with a median usage of 2 h and 22 min per night. The effect of age on CPAP adherence has not fully been studied; May et al. have reported recently that increasing age is associated with improved adherence in a study population < 65 years [9].

The purpose of this study is to evaluate CPAP adherence in patients > 70 years compared with younger patients < 50 years.

## Methods

### Subjects

Patients were recruited during the years 2017–2018 at our sleep unit. All subjects had a respiratory event index (REI) ≥ 5 events/h.

There is a current tendency to progressively increase the retirement age to over 65 years. Therefore, we included in the senior group only patients > 70 years. The maximum age was set at 100 years.

For the control group, we included junior patients (aged between 18 and 50 years) who were CPAP naïve.

All patients provided written informed consent. The ethical committee of Helsinki University Hospital approved the study protocol (TMK11§101/29.4 2015, code 61/13/03/01/2015).

We excluded patients with previous CPAP experience, non-cooperative subjects, and subjects suffering from obesity hypoventilation syndrome, central apnea, severe disease with life expectancy < 1 year, or severe and unstable psychiatric disorder.

All patients underwent a sleep study with a type-3 cardiorespiratory device according to the AASM classification, NOX T3 (Noxturnal, Iceland). We applied the AASM criteria for apnea and hypopnea [10].

We applied the same criteria for CPAP initiation as described previously [11] (REI ≥ 15 events/h or REI ≥ 5 events/h if the patient had daytime hypersomnolence or significant comorbidities).

#### CPAP initiation

Patients starting CPAP underwent a 1-h familiarization session at the sleep clinic with the CPAP device and masks as described previously [12]. Two to three months after CPAP initiation, patients were also examined to ensure good therapy response. Thereafter, follow-up contacts were routinely planned annually. All patients used the same CPAP device (Resmed, San Diego, CA). CPAP data were downloaded using Rescan program.

We collected the following data at baseline (before CPAP initiation): gender, BMI, sleep study parameters, Epworth Sleepiness Scale (ESS), and comorbidities. Each patient also underwent cognitive and physical tests.

All patients were evaluated at 1 year from CPAP initiation. Data on CPAP, ESS, and cognitive and physical tests were collected at this time.

### Cognitive and physical test

#### The Nine-Hole Peg Test

The Nine-Hole Peg Test (NHPT) is a simple, rapid assessment for finger dexterity, also known as fine manual dexterity [13]. The patient is requested to take the pegs from a container, one by one, and place them into the holes on the board. The patient then places the pegs one by one back into the container as quickly as possible.

**Fig. 1:**
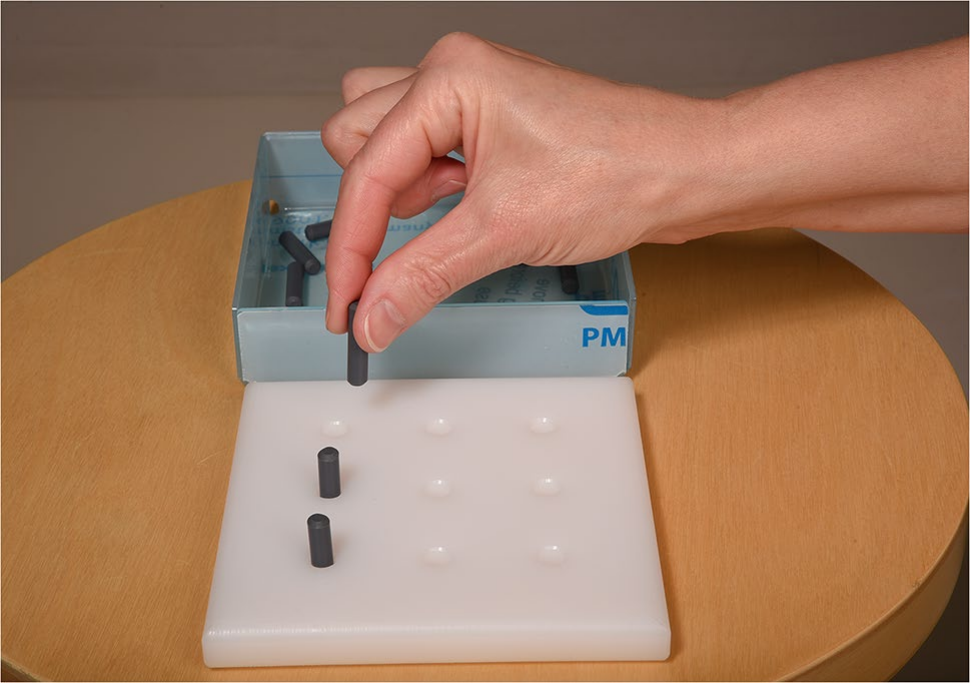
Nine-Hole Peg Test. The patient is requested to take the pegs from a container, one by one, and place them into the holes on the board. The patient then places the pegs one by one back into the container as quickly as possible

Participants must remove the pegs from the holes, one by one, and place them back into the container. The test is performed twice, first by the dominant hand (Fig. 1).

### Pinch strength testing

**Fig. 2:**
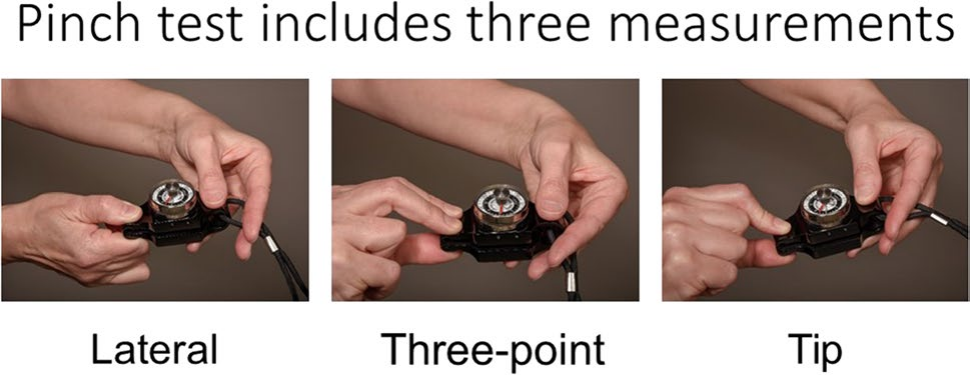
Pinch test. In the pinch test, the patient pinches the measurement device in three different positions as tightly as possible

A pinch grip is a form of precision grip whereby an object is pinched in three ways (lateral pinch, three-point pinch, and tip pinch) [14] (Fig. 2).

Three trials of each of the following pinch positions were performed twice. We retained the best value of each position.

1. Lateral pinch: The pinch meter is positioned between the thumb pulp and the lateral aspect of the middle phalanx of the index finger.
2. Three-point pinch: The pinch meter is positioned between the subject’s thumb pulp and the pulps of the index and middle fingers.
3. Tip pinch: The pinch meter is positioned between the tip of the thumb and tip of the index.

All measurements were also performed for the non-dominant hand.

### Measurements of shoulder movement range (figure movement)

**Fig. 3:**
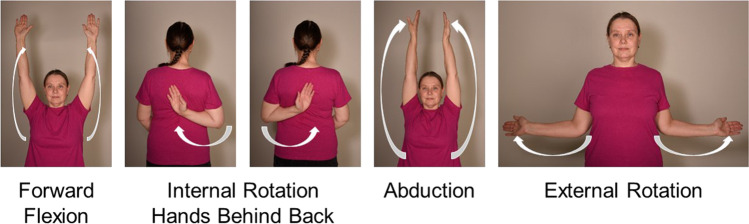
Shoulder movements. The nurse measures the movement angles of the patient’s upper limb in different positions

For forward flexion measurement, the straight arm is raised in front of the body, with the palm down, as high as possible. For abduction, the straight arm is raised at the side, with the palm down, as high as possible. For external rotation, the elbows are held by the sides of the body and bent at 90° with palms facing each other. With the elbows in contact with the body, the hands are then spread outwards as far as possible. For internal rotation, the arm is put behind the back with the elbow bent. The subject reaches as far up the back as possible. This distance is measured from a specific point on the spine [15] (Fig. 3).

### Statistics and sample size

This was a non-inferiority study. We hypothesized that CPAP daily use in senior patients during the first year of follow-up is not inferior to CPAP daily use in junior patients; namely, senior patients use the CPAP device daily at least as often as junior patients do. The mean daily CPAP use was estimated at 4 ± 2 h. We set the margin of non-inferiority at 1 h of CPAP use, α-value at 0.05 and β-value at 0.1 for one-sided test. The number of subjects needed was 69 for each group.

We estimated sample size by using Clincalc.com (https://clincalc.com/stats/samplesize.aspx). Analysis of variance, chi-square test, and *t* test were used when appropriate. P < 0.05 was considered statistically significant. Normal distribution was checked using Kolmogorov–Smirnov and Shapiro–Wilk tests. We also calculated skewness and kurtosis values. For non‐parametric continuous variables without normal distribution, we used the Mann–Whitney U test to compare two samples and reported data as median and lower to upper quartile. We used SPSS (version 25; IBM, Armonk, New York) to compute differences in demographic, clinical, and measured variables. A Bonferroni correction for multiple comparison was used when necessary.

## Results

We included 72 patients in the senior group; of these, 4 patients refused CPAP initiation after initial acceptance. One patient did not attend the 1-year follow-up visit. Fourteen patients discontinued CPAP therapy within 1 year and 53 continued therapy beyond 1 year.

**Fig. 4:**
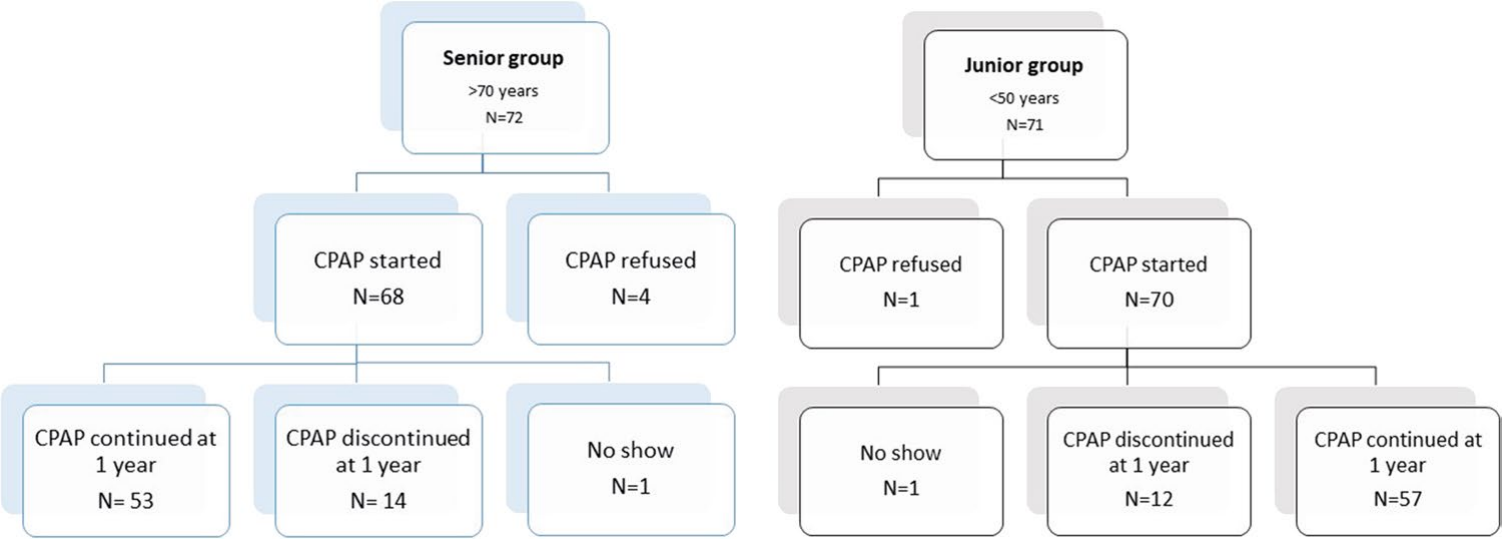
Patient flowchart

We included 71 patients in the junior group; of these, 1 refused to initiate CPAP therapy after initial acceptance. One patient did not attend the 1-year follow-up visit, 12 patients discontinued CPAP therapy within 1 year, and 57 continued CPAP therapy (Fig. 4).

No significant differences between senior and junior groups were observed in baseline values of BMI, AHI, AHI supine, ODI3, or ESS. In the senior group, the percentage of women (49%) was significantly higher than in the junior group (24%). Moreover, mean SpO_2_, wake SpO_2_, and cumulative time below 90% (CT 90%) were significantly poorer in the senior group than in the junior group. The incidence of coronary heart disease (MCC) and hypertension was significantly higher in senior patients than in junior patients (Table 1).

**Table 1:** Patient charachteristics

	Seniors	Juniors	P
Number	72	71	
Women, number (%)	35 (49)	17 (24)	0.003
Age, year, mean (SD)	75 (4)	40 (8)	0.000
Body mass index, BMI baseline, mean (SD)	31 (8)	31 (5)	0.996
Respiratory event index, REI, baseline, mean (SD)	34 (17)	37 (23)	0.390
REI supine baseline, mean (SD)	47 (19)	52 (28)	0.205
Oxygen desaturation index, ODI3 baseline, mean (SD)	32 (18)	38 (23)	0.126
Pulse oximetry, SpO2 mean (SD)	92 (2)	93 (2)	0.000
SpO2 wake, mean (SD)	96 (1)	97 (1)	0.000
Cumulative time, CT 90% baseline, mean (SD)	23 (25)	13 (16)	0.009
Epworth Sleepiness Scale ESS, mean (SD)	7.7 (4.8)	9.9 (3.2)	0.227
Hypertension, number (%)	49 (68)	14 (20)	0.000
DM number (%)	23 (32)	8 (11)	0.002
Asthma, number (%)	5 (7)	6 (9)	0.500
Coronary heart disease, MCC, number (%)	16 (22)	1 (1)	0.000
Alzheimers disease, number (%)	2 (3)	0 (0)	0.252
Rheumathoid arthritis, number (%)	5 (7)	2 (3)	0.226
Chronic obstructive pulmonary disease, number (%)	4 (6)	1 (1)	0.187
Chronic rhinitis, number (%)	1 (1)	8 (11)	0.016
Still on CPAP at 1 year (%)	53	57	0.428

### CPAP acceptance and daily use

**Fig. 5:**
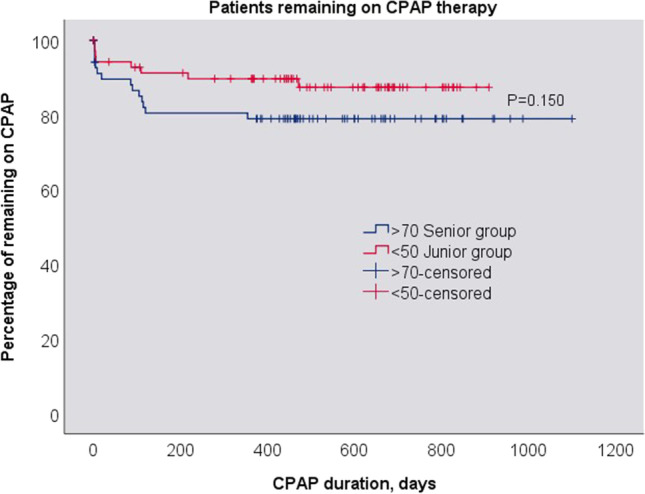
Kaplan-Meyer plot of proportions of senior and junior patients remaining on CPAP

**Fig. 6:**
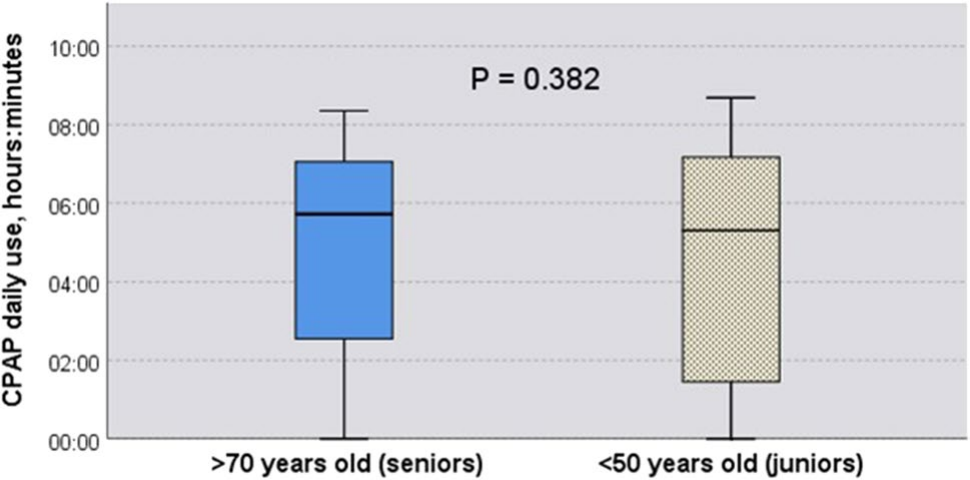
CPAP daily use. No significant difference in mean CPAP daily use between senior and junior patients was observed

We did not observe any statistically significant difference in the percentage of patients continuing therapy beyond 1 year between the senior and the junior groups (X2 (1, N = 143) = 0.896 (p = 0.428)). No differences were observed up until the last control visit at 1100 days (Fig. 5). No significant difference in daily CPAP use was found between senior and junior patients (4:53 ± 2:44 hh:min vs. 4:23 ± 3:00 hh:min) (F (1, 129) = 1.001; (p = 0.319)) (Fig. 6).

### MMSE

No significant difference was observed in MMSE values between senior and junior patients (median 27.00, range 15–30 vs. 28.00, range 21–30).

### Nine-Hole Peg Test

The senior group was significantly slower both in the dominant and non-dominant hand than the junior group, Table 2.

**Table 2:** Values of dominant upper extremity movements and pinch test

		Senior	Juniors		Senior	Juniors		Senior	Juniors		Senior	Juniors	
Shoulder movements		Forward flexion		P	Abduction		P	External rotation		P	Internal rotation		P
Mean, angle in degree °		177	179		177	179		64	73		26	22	
Median		180	180	0.002	180	180	0.700	70	80	0.028	26	22	0.023
Percentiles	25	180	180		180	180		45	55		22	18	
	75	180	180		180	180		80	90		30	27	
Pinch test		Tip			Lateral			Three-point					
Mean	lbs	12	15		19	24		16	20				
Median		12	15	0.000	18	25	0.000	15	20	0.000			
Percentiles	25	10	13		16	21		13	18				
	75	15	18		22	28		19	24				

### Pinch test

The pinch test values of senior patients were significantly lower than those of junior patients, both in the dominant and in the non-dominant hand, Table 2.

### Shoulder movement

As expected, most of the shoulder movements were more restricted in the senior patients than in the junior patients, Table 2.

## Discussion

The main finding in this study was that there is no difference in CPAP adherence at 1 year between senior patients with OSA aged > 70 years and junior patients aged < 50 years. This has a significant clinical implication when evaluating therapy options for senior patients suffering from sleep apnea. The number of patients abandoning CPAP therapy in the senior group was not statistically different from that of the junior group.

Our results are consistent with that of McMillan [2] and Roche et al. [16], who recommended CPAP therapy routinely to senior patients with sleep apnea. Our patients used CPAP therapy 4 h a day, whereas those reported by MacMillan et al. used CPAP for 2 h a day. Even with low daily CPAP use, they reported reductions in ESS scores. Our baseline mean ESS scores were not high and did not change significantly with CPAP therapy. ESS scores do not correlate with daytime hypersomnolence [17] and may not predict CPAP success [2]. We do not have a rational explanation for these CPAP outcomes. It is known that CPAP adherence is influenced by factors such as diagnostic methods, symptoms, psychological profile, CPAP initiation information, modalities, and follow-up. Further studies are needed on senior patients.

Memory loss may interfere with therapy adherence [5]. Our patients had no significant memory loss as measured by MMSE.

Mobility and physical tests did not affect CPAP adherence. This situation would have been different a decade ago, when CPAP devices and interfaces were bulky and cumbersome.

The strengths of our study include a long follow-up period (1 year), a relatively large number of subjects, and a very low dropout percentage. However, there are also several limitations. This was a single-center study. No quality-of-life questionnaires were used to evaluate the effect of CPAP therapy. We did not measure the effect of CPAP therapy on reducing cardiovascular risks. None of our patients had signs of severe Alzheimer’s disease. The basic insurance system in Finland covers all subjects regardless of working status and this may differ from other countries.

The percentage of women in the senior group was significantly more than in the junior group. This reflects the population of Finland, where the proportion of women increases with age [https://www.stat.fi/tup/suoluk/suoluk_vaesto_en.html]. We previously reported that gender did not interfere with CPAP adherence in OSA patients without previous CPAP experience [9].

## Conclusions

Adherence to CPAP therapy was similar between patients > 70 years and < 50 years. Advanced age was not an obstacle to good CPAP adherence.

## Abbreviations

AHI-CPAP: Apnea hypopnea index reported by the CPAP device (residual AHI)
BMI: Body mass index
CPAP: Continuous positive airway pressure
CT 90%: Cumulative time with SpO2 below 90%
ESS: Epworth Sleepiness Scale
MCC: Coronary heart disease
ODI3: Oxygen desaturation index of 3%
OSA: Obstructive sleep apnea
REI: Respiratory event index
SpO2: Pulse oxygen saturation
